# Supplementary material for: Haemoglobin polymorphism in different blood levels of Ethiopian Zebu x Holstein Friesian crossbred dairy cattle
Source: PLoS One. 2026 Mar 25;21(3):e0340009. doi: 10.1371/journal.pone.0340009 (PMC13016309; doi:10.1371/journal.pone.0340009)
Supplement: Attachment — Submitted filename: Corection PONE-D-25-40346.docx [file pone.0340009.s002.docx]

PONE-D-25-40346

Haemoglobin polymorphism 1 in different blood levels of Ethiopian Zebu x Holstein Friesian crossbred dairy cattle

Abstract

1. Line 24–25: Misinterpretation of FIS values. FIS= 0.38 and 0.41 are described as “inbreeding coefficient value of the HF crosses relative to the subpopulations grouped into…”, but FISis not a single value per grouping type—it should be one estimate per subpopulation or an average, not two separate values for “blood levels” and “locations” without clarifying they are separate analyses.

2. Line 26–27: States FST = 0.01 for location and 0.06 for blood level, implying higher genetic differentiation among blood levels—but FST = 0.06 is still very low (near panmixia), yet the phrasing may mislead readers into overinterpreting biological significance.

3. Line 29–30: Concludes a “relatively higher level of similitude” based on low heterozygosity and high homozygosity—this is contradictory: high homozygosity suggests low genetic variation, not necessarily “similitude” across groups. The term is vague and scientifically imprecise.

Introduction

4. Line 57: Calls hemoglobin an “evergreen red protein”— non-scientific, metaphorical language inappropriate for a research manuscript.

5. Line 68–69: States only “a few research works” on Hb polymorphism in Ethiopia, citing Gezahegn (1996) and Pal & Mummed (2014)—but Pal & Mummed (2014) studied Ogaden cattle, not general Ethiopian breeds. This misrepresents scope.

6. Line 77–78: Asserts protein polymorphism studies are “easier to implement” than DNA-based methods—but this is context-dependent and outdated (e.g., PCR-based genotyping is now cheaper and more accessible than electrophoresis in many settings).

7. Line 85–86: Hypothesis states Hb frequencies “are under Hardy-Weinberg equilibrium”—but HWE is a null model, not a hypothesis to be confirmed; appropriate phrasing would be testing deviation from HWE.

Materials and Methods

8. Line 120–122: Claims 13 cows per blood level per location → 3 blood levels × 3 locations × 13 = 117, which is correct—but later tables report genotype counts that don’t sum correctly (e.g., Table 1: HF87.5% shows 38+1+0 = 39, but 13 per location would be 39 total—OK), yet Table 2 totals per location (e.g., SHA: 35+3+1=39) seem consistent, so this is not an error, but potential confusion arises.

9. Line 140–143: Uses carbon tetrachloride (CCl4) for hemolysate prep—CCl4 is highly toxic, carcinogenic, and largely obsolete in modern labs. No safety or ethical justification provided despite known hazards.

10. Line 147–148: Uses agarose gel electrophoresis at pH 8.6 for Hb typing—standard practice uses starch or cellulose acetate gels, not agarose, for Hb isoform separation. Agarose has poor resolution for small charge differences in globins—methodologically questionable.

11. Line 155–157: Assigns fast band = HbBB, slow = HbAA—but in cattle, HbB is typically the slow-migrating variant (contrary to human HbS). Literature (e.g., Bachmann et al. 1978) shows HbB (Zebu) migrates slower than HbA (taurine). Likely misassignment of allele identity.

Results

12. Table 1, HF87.5% row: Expected HbBB = 0, observed = 0—but Chi-square value listed as “1.0ns”—this is mathematically impossible if expected = observed = 0. Likely a software or transcription error.

13. Table 1, Entire population: Observed HbAB = 13, expected = 22.4 → large deviation, Chi-square = 0.00 (p < 0.001)—but 0.00 is not a valid Chi-square value; probably means p = 0.00, but mislabeled.

12. Table 3: Expected heterozygosity (He) for HF50% = 0.32, Observed (Ho) = 0.18 → deficit of heterozygotes, consistent with inbreeding—but no statistical test (e.g., FIS per group) is provided in table, only pooled.

13. Table 5: Genetic identity >1.0 (e.g., HF50% vs HF50% = 1.0, OK—but HF75% vs HF75% = -0.99? Negative identity? Impossible—values must be 0–1. Typo: likely 0.99, not -0.99.

14. Table 6: SHA vs SHA = -1.0—again, identity cannot be negative. Should be 1.0. Formatting error (hyphen misread as minus).

15. Line 263–265: Reports FST= 0.01 (locations) as “significant” and FST= 0.06 (blood levels) as “non-significant”—but no p-values or confidence intervals provided to support “significance” claims.

Discussion

16. Line 294–295: Claims “highest frequency HbB was observed in HF50%”—but Table 1 shows HbB freq = 0.19 in HF50%, 0.12 in HF75%, 0.01 in HF87.5%—so correct, but then implies HbB is Zebu-associated, which aligns with literature. However, if allele labeling is inverted (see point 12), this conclusion collapses.

17. Line 306–307: Links HbA frequency to altitude—but Shashemene (1900–1950 m), Hawassa (1750 m), Dilla (1300–2500 m)—yet Dilla has lowest HbA (0.85) vs SHA (0.94). Contradicts the claim.

18. Line 316–317: States “average heterozygosity of 0.1”—but Table 3 shows pooled Ho = 0.11, close—but then compares to “recommended range 0.3–0.8”— no citation for this arbitrary “recommended” range. Genetic diversity expectations depend on species and marker type; for single-locus protein markers, Ho < 0.2 is common.

References

19. Reference formatting inconsistencies:

- Braend (1972): DOI `10.5555/19730102670` is invalid.

- Gebrehiwet (2020): DOI `10.5555/20203476252` similarly invalid.

- Takezaki and Nei (1996): Journal name shortened incorrectly; should be Genetics, not Genet.

→ Suggests inadequate reference validation.
