# Supplementary material for: Haemoglobin polymorphism in different blood levels of Ethiopian Zebu x Holstein Friesian crossbred dairy cattle
Source: PLoS One. 2026 Mar 25;21(3):e0340009. doi: 10.1371/journal.pone.0340009 (PMC13016309; doi:10.1371/journal.pone.0340009)
Supplement: Attachment — Submitted filename: Recommendation PONE-D-25-40346.docx [file pone.0340009.s003.docx]

PONE-D-25-40346

Haemoglobin polymorphism 1 in different blood levels of Ethiopian Zebu x Holstein Friesian crossbred dairy cattle

Summary of Critical Errors:

- Methodological: Use of agarose gel (unsuitable for Hb), toxic CCl 4, incorrect Hb migration interpretation.

- Statistical: Inappropriate Chi-square use, missing p-values, impossible genetic identity values.

- Conceptual: Misuse of HWE hypothesis, vague terms like “similitude”, unsupported “recommended heterozygosity” range.

- Data reporting: Table inconsistencies (negative identity, zero expected counts).

- Ethical/technical: Safety oversight, reference errors, ambiguous consent.

These issues undermine the reliability of conclusions and suggest the need for major revision before publication.
